# TiO_2_ nanotubes and mesoporous silica as containers in self-healing epoxy coatings

**DOI:** 10.1038/srep38812

**Published:** 2016-12-12

**Authors:** Poornima Vijayan P., Mariam Ali S. A. Al-Maadeed

**Affiliations:** 1 Center for Advanced Materials, Qatar University, P.O. Box 2713, Doha, Qatar; 2 Materials Science and Technology Program, Qatar University, P.O. Box 2713, Doha, Qatar

## Abstract

The potential of inorganic nanomaterials as reservoirs for healing agents is presented here. Mesoporous silica (SBA-15) and TiO_2_ nanotubes (TNTs) were synthesized. Both epoxy-encapsulated TiO_2_ nanotubes and amine-immobilized mesoporous silica were incorporated into epoxy and subsequently coated on a carbon steel substrate. The encapsulated TiO_2_ nanotubes was quantitatively estimated using a ‘dead pore ratio’ calculation. The morphology of the composite coating was studied in detail using transmission electron microscopic (TEM) analysis. The self-healing ability of the coating was monitored using electrochemical impedance spectroscopy (EIS); the coating recovered 57% of its anticorrosive property in 5 days. The self-healing of the scratch on the coating was monitored using Scanning Electron Microscopy (SEM). The results confirmed that the epoxy pre-polymer was slowly released into the crack. The released epoxy pre-polymer came into contact with the amine immobilized in mesoporous silica and cross-linked to heal the scratch.

The self-healing concept has been studied to develop effective corrosion protection coatings for metals^1^,^2^,^3^,^4^,^5^. The self-healing ability of coatings without manual intervention extends the service life of the coatings, leading to a significant reduction in the maintenance cost for marine assets, oil and gas pipe lines and aerospace materials. Different types of micro- or nano-containers are used for the storage and release of corrosion inhibitors or self-healing agents, depending on if the specific corrosion triggering conditions are dependent upon chemical changes (e.g., on pH change) or upon mechanical damage^6^. Corrosion inhibitors or healing agents are usually immobilized or encapsulated inside polymeric micro capsules^7^,^8^,^9^,^10^ and inorganic meso- and nano-porous materials^2^,^11^,^12^,^13^.

The self-healing in epoxy systems based on the encapsulation of the epoxy and the curing agent in containers provides a repair system that shares chemical properties with the host epoxy^9^. Though the epoxy encapsulation is readily achievable, the encapsulation of the liquid amine curing agent is difficult because of its high reactivity. Few studies have been reported where an effective encapsulation of amine curing agent was achieved. The vacuum infiltration of a reactive amine (diethylenetriamine) curing agent into hollow poly (urea-formaldehyde) (PUF) microcapsules was accomplished by Jin *et al*.^14^.

Inorganic particles with nanocavities have a large surface area, a high pore volume and a low density and stability favorable for the storage of the healing agents. Additionally, the use of inorganic nanomaterials as reservoirs for healing agents can eliminate the tedious encapsulation process. Previously, inorganic particles such as cerium molybdate^15^, mesoporous silica^13^,^16^, hollow titanium dioxide spheres^2^, cerium titanium oxide^17^, etc., were reported as containers for corrosion inhibitors to provide self-healing protective coatings for metals. Fibrous and tube shaped materials including cellulose nanofibers^18^,^19^, clay nanotubes^20^,^21^,^22^, etc., have also been reported as effective containers for corrosion inhibitors. The healing agents inside these hollow structures are released upon formation of the cracks and “are only released in close proximity of coating defects”^13^,^22^. The inherent anti-corrosion ability of the nanomaterials based on these inorganic compounds is an added advantage in this type of metal coatings^23^,^24^,^25^.

This research aims to explore the potential of TiO_2_ nanotubes and mesoporous silica as containers to store epoxy pre-polymer and the amine curing agent, respectively. A simple method for the preparation of self-healing epoxy coating for carbon steel by utilizing both mesoporous silica and TiO_2_ nanotube was used in this study. TiO_2_ nanotubes (TNT) prepared by a hydrothermal method were used to encapsulate the epoxy pre-polymer. At the same time, amine curing agent was immobilized in high surface area mesoporous silica (SBA-15). The encapsulation of the epoxy monomer in the TiO_2_ nanotubes and the distribution of mesoporous silica on the composite coating of the filled TiO_2_ nanotubes were investigated using TEM analyses. The self-healing ability of this two-component self-healing epoxy coating was evaluated using electrochemical impedance spectroscopy (EIS) and Scanning Electron Microscopy (SEM).

## Methods

### Materials

Poly(ethylene glycol)-block-poly(propylene glycol)-block-poly(ethylene glycol) (EO20PO70EO20) (PEG-PPG-PEG, Pluronic^®^ P-123) with an average Mn-5,800 supplied by Sigma–Aldrich was used as template for the synthesis of mesoporous silica. Tetraethyl orthosilicate (TEOS) (reagent grade 98%) from Sigma–Aldrich was used as the silica source. TiO_2_ (anatase) powder supplied by Sigma-Aldrich was used as the precursor for TiO_2_ nanotubes. Hydrochloric acid (HCl), ethanol and sodium hydroxide pellets used were supplied by Sigma–Aldrich.

Epon 826 and diethylene triamine curing agent (Epikure 3223), purchased from Miller-Stephenson Chemical Co., USA, were used for coating. Epon 815C is a form of Epon 826 diluted with butyl glycidyl ether and was inserted inside the TiO_2_ nanotubes. Polished carbon steels were used as the substrate for coating.

### Characterization techniques

The morphology of the synthesized nanomaterials was studied using scanning electron microscopic (SEM) and transmission electron microscopic (TEM) techniques. Nova NanoSEM field emission scanning electron microscopy with an accelerating voltage of 5.0 kV was used. The TEM study was conducted using an FEI TECNAI GF20 S-TWIN electron microscopy instrument. The sample was prepared by dispersing the final powders in water and dropping the dispersion onto carbon copper grids. The morphology of the epoxy composite coating was also studied using TEM. Approximately 100-nm-thick sections were microtomed at room temperature using a diamond knife and transferred to carbon-Cu grids.

Brunauer–Emmett–Teller (BET) surface area analysis of the nanomaterials was performed using the Micromeritics Chemisorb 2750 pulse chemisorption system. The sample mass was approximately 0.04 g. The sample was degassed in the presence of pure nitrogen for 30–45 min at 200 °C prior to analysis. The BET adsorption/desorption isotherm was determined by nitrogen sorption from a mixture of helium (70%)/nitrogen (30%) gas at the temperature of liquid nitrogen (77 K).

The wettability of the coated carbon steel was evaluated using water contact angle measurements. The contact angle measurements were conducted in a Kruss G40 contact angle goniometer at room temperature following the sessile drop principle. Electrochemical impedance spectroscopic (EIS) studies of the coatings were conducted using a Gamry Reference 600 Potentiostat/Galvanostat/ZRA. An Ag/AgCl electrode was used as the reference electrode with a stainless steel counter electrode arranged parallel to the exposed sample to complete the cell. A 3.5 wt% NaCl aqueous solution was used as the electrolyte solution. The sample was in contact with the electrolyte solution, and the measurements were performed at ambient temperature. EIS measurements were performed over the frequency range of 0.01–100,000 Hz. SEM images of the scratched coating surfaces were taken at defined intervals using Benchtop scanning electron microscopy (JCM 6000 – Jeol) to observe the self-healing process.

### Synthesis and characterization of mesoporous silica (SBA-15)

**Figure 1:**
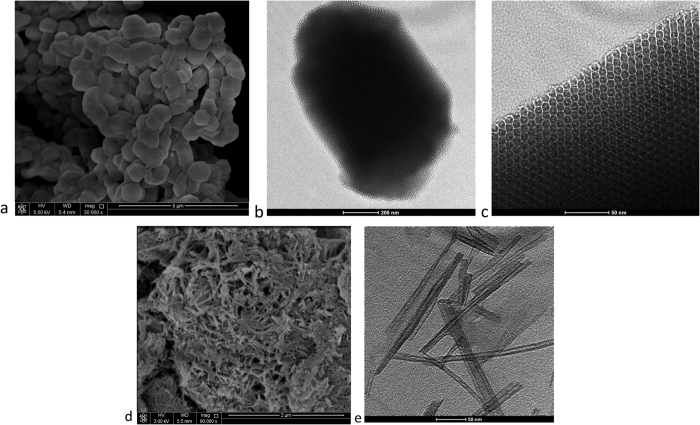
(**a**) SEM image of mesoporous SBA-15 particles, TEM image of (**b**) mesoporous SBA-15 single particle, (**c**) details of (**b,d**) SEM and (**e**) TEM images of TiO_2_ nanotubes.

SBA-15 mesoporous silica was synthesized using the amphiphilic triblock copolymer poly(ethylene glycol)-block-poly(propylene glycol)-block-poly(ethylene glycol) (EO20PO70EO20) as described in ref. 26. A total of 4 g of the amphiphilic triblock copolymer was dispersed in a solution of 30 g of water and 120 g of 2 M HCl, and the mixture was stirred for 5 h. Tetraethyl orthosilicate (TEOS; 9.5 g) was then added to the homogeneous solution and stirred. The resulting gel was aged at 35–40 °C for 24 h and subsequently heated to 100 °C for 18 h. After cooling to room temperature, the precipitate was washed with distilled water and ethanol to remove the copolymer. The product was dried at room temperature for 24 h and then calcined at 550 °C for 6 h to decompose the triblock copolymer. Figure 1a shows the SEM images of synthesized SBA-15 mesoporous silica. The particles are uniform in shape with an average particle size of 500 nm. TEM image of a single mesoporous silica particle is shown in Fig. 1b. A detailed TEM image (Fig. 1c) of a single particle clearly shows the well-ordered hexagonal arrangement of cylindrical mesopores within the particle. The calculated average pore diameter of the cylindrical pore is 4 nm. The BET surface area of SBA-15 mesoporous silica is 365.77 m^2^/g.

### Synthesis and characterization of TiO_2_ nanotubes (TNT)

A hydrothermal method was used to synthesis TiO_2_ nanotubes^27^. First, 1.2 g of TiO_2_ powder was mixed with 20 ml of 10 N NaOH solution in a Teflon beaker and vigorously stirred for 15 min. The mixture was then transferred to a Teflon-lined autoclave and heated in a preheated oven at 130 °C for 10 h. The obtained precipitate was washed with distilled water. The washed precipitate was dipped in 0.1 M HCl solution for 30 min and washed again with HCl solution and distilled water until a solution pH of 7 was achieved. Finally, the synthesized powder was dried. The SEM and TEM images of the prepared TiO_2_ nanotubes are shown in Fig. 1d and e, respectively. The average tube diameter was 10 nm. The BET-determined surface area of the prepared TiO_2_ nanotubes is 173.93 m^2^/g.

### Encapsulation of the epoxy pre-polymer into TiO_2_ nanotubes (TNT)

**Figure 2:**
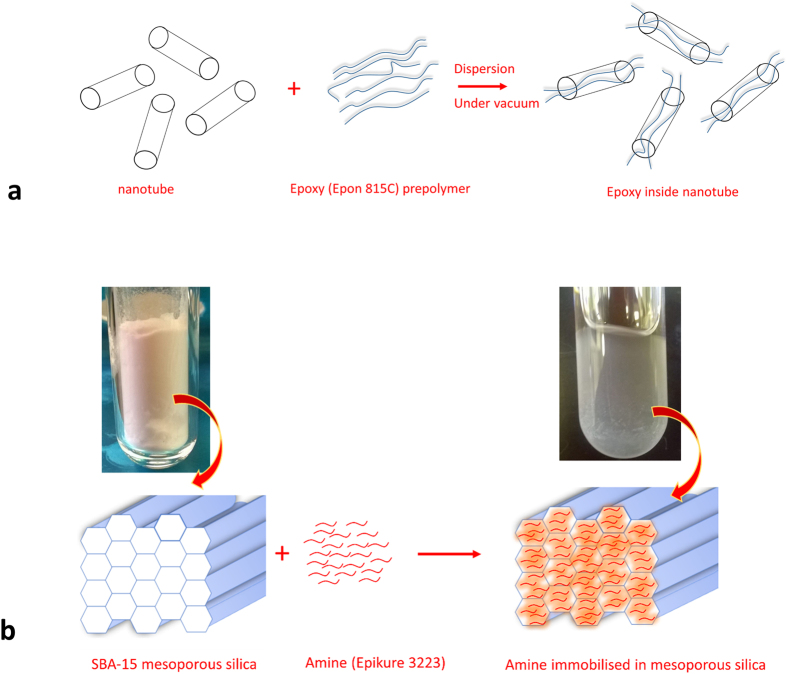
Schematic diagram showing (**a**) the insertion of epoxy monomer inside the TiO_2_ nanotube cavity and (**b**) the immobilization of amine curing agent in the pores of SBA-15.

Epon815C/15 phr TNT solution was prepared by stirring for 30 min at 1000 rpm. The obtained homogenous solution was placed inside a vacuum jar for 24 h to complete the encapsulation of the epoxy inside the TiO_2_ nanotube. During this process, the air trapped inside the tube lumen was replaced by the epoxy pre-polymer. A schematic illustration of insertion of epoxy monomer into the TiO_2_ nanotube cavity is shown in Fig. 2a.

### Immobilization of the amine curing agent in mesoporous silica

The prepared mesoporous silica was stirred with an excess of Epikure 3223. The mixture was incubated for 24 h at 25 °C with moderate shaking to immobilize the amine molecules in pores of SBA-15. The immobilization of amine curing agent in the pores of SBA-15 is shown in Fig. 2b.

### Preparation of self-healing coating on carbon steel

The epoxy encapsulated Epon815C/15 phr TNT solution was diluted with Epon 826 to make epoxy/7.5 phr TNT solution. This homogenous solution was then mixed with 0.8 phr curing agent immobilized in mesoporous silica. The encapsulated TNT to curing agent immobilized mesoporous silica ratio was fixed based on stoichiometric ratio of epoxy to curing agent used in this study. The stoichiometric ratio of epoxy: diethylene triamine curing agent is 100:11 (For 100 g epoxy 11 g of curing agent). Similarly, the encapsulated TNT: curing agent immobilized mesoporous silica ratio was fixed as 7.5 phr: 0.89 phr. Next, 11 phr curing agent was added to the mixture and was degassed by sonication for 10 min. The mixtures were then coated on one side of the carbon steel to obtain a coating with an average thickness of 300 μm. The coating was allowed to cure for 24 h. The control epoxy coatings were prepared without TNT and mesoporous silica. The control epoxy coating is abbreviated as ‘EP’ and the epoxy coating loaded with epoxy pre-polymer encapsulated TNT and curing agent immobilized mesoporous silica is denoted as ‘EP/TNT/Imeso’. The abbreviated names are used for the coating samples in the subsequent text.

## Results and Discussion

### Quantitative determination of the amount of nanotube filled with the epoxy

The approach adopted by Suzuki *et al*.^28^,^29^ was used to quantitatively calculate the amount of TiO_2_ nanotube loaded with the epoxy monomer. In this approach, the degree of nanotube filled with the epoxy monomer in the composite was calculated in terms of ‘dead pore ratio’ using the equations (1) and (2). Because the amount of amine immobilized mesoporous silica in the EP/TNT/Imeso composite is very low (0.8 phr) compared to the amount in the TiO_2_ nanotube (7.5 phr), the presence of mesoporous silica is not considered while calculating the dead pore ratio.

The vacant volume (V) (not filled with monomer) per weight (g) of loaded TNT in the EP/TNT/Imeso composite coating is given by


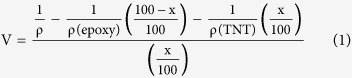



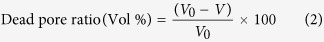


where V_0_ is the total pore volume of the TNT (0.2784 cm^3^/g), X is the additive amount ratio in wt % of the TiO_2_ nanotube in the composite (6.3291), and ρ, ρ (epoxy) and ρ(TNT) are the density of the composite, epoxy polymer and TNT, respectively. (ρ(TNT) − 4.23 g/cm^3^, ρ(epoxy) −1.1983 g/cm^3^ and ρ −1.2775 g/cm^3^).

The calculated dead pore ratio for the EP/TNT/Imeso coating composite was 94%. A 100% dead pore ratio represents that all the nanotube pores are filled with the polymer, and a 0% dead pore ratio indicates that all the pores are empty. A 94% dead pore ratio value of the EP/TNT/Imeso coating composites indicates that the pores are satisfactorily filled with epoxy.

### Morphology of the coating composite

**Figure 3:**
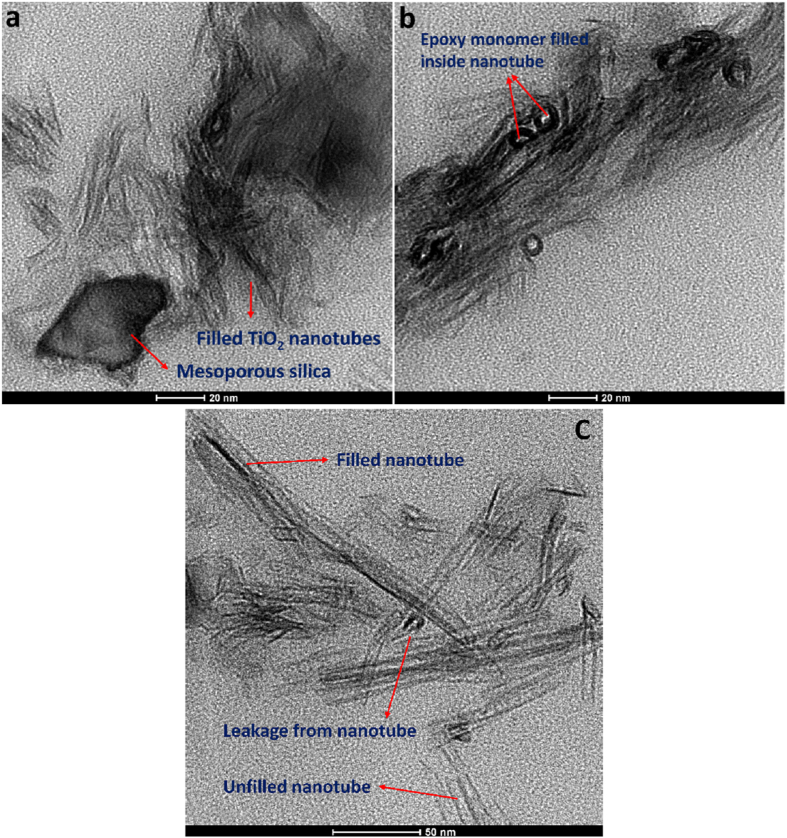
TEM images of EP/TNT/Imeso coating composite.

Figure 3 shows the TEM images of the EP/TNT/Imeso coating composite. The amine immobilized mesoporous silica particles are found to be randomly distributed among the groups of TiO_2_ nanotubes (Fig. 3a). The diameter of the mesoporous silica particles in the TEM images of the coating is seen to be smaller than that of the bare mesoporous silica particle (as seen in Fig. 1b). This is due to the fact that the portion of the mesoporous silica particle present in the TEM section might be from the edges of the hexagonal particle. The TEM images show that most of the TiO_2_ nanotubes are filled with epoxy monomer. The edges of the TiO_2_ nanotubes cut by microtome sectioning are shown as black circles in the TEM images (Fig. 3b). Inside these black circles, the epoxy monomers are shown as black dots. The TEM image (Fig. 3c) also demonstrates the leakage of the epoxy monomer from some of the nanotubes that were cut during microtome sectioning. Some of the TiO_2_ nanotubes are found to be empty.

### Wettability of the coatings

**Figure 4:**
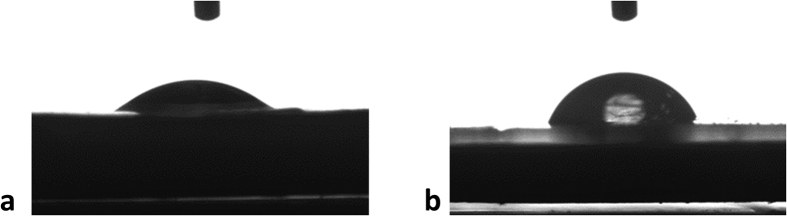
Contact angle of (**a**) control epoxy and (**b**) EP/TNT/Imeso coatings.

The wettability of the prepared coatings was evaluated using contact angle measurements. The measured contact angles for the control epoxy and EP/TNT/Imeso coatings are 32.47° and 64.30°, respectively (Fig. 4). The presence of encapsulated TiO_2_ nanotubes and mesoporous silica makes the EP/TNT/Imeso coating less hydrophilic. The rougher surface created by the presence of the nanomaterials reduces the hydrophilicity of the coating, thereby improving its waterproof properties.

### Electrochemical impedance spectroscopic (EIS) analysis

**Figure 5:**
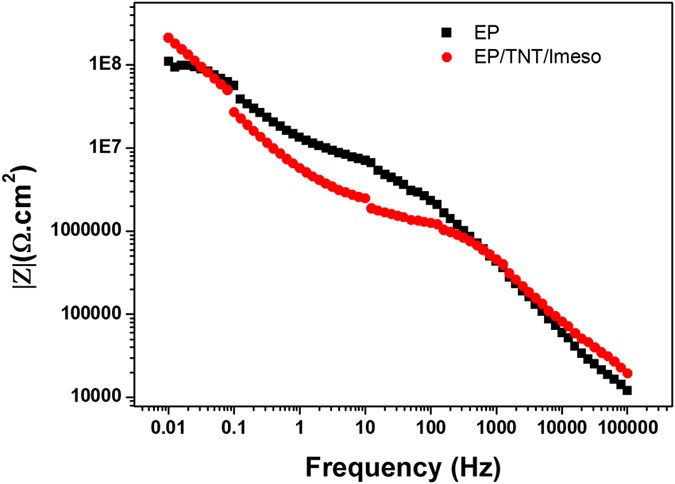
EIS Bode diagrams for unscratched control epoxy coating (EP) and EP/TNT/Imeso in 3.5 wt% NaCl solution.

Electrochemical impedance spectroscopy is an appropriate technique for the investigation of the anticorrosion and self-healing abilities of coatings^30^,^31^. Initially, the corrosion responses of the control epoxy and EP/TNT/Imeso coatings were compared. The EIS bode plot of the control epoxy coating and the EP/TNT/Imeso coating is shown in Fig. 5.

The impedance values measured in the low frequency range can be used to assess the corrosion resistance of the system^3^,^32^. As shown in Fig. 5, the impedance value at 0.01 Hz for the control epoxy coating is 1.101E8 Ω.cm^2^, and that of EP/TNT/Imeso is 2.132E8 Ω.cm^2^. A higher low frequency impedance value for the EP/TNT/Imeso coating compared to that of the EP coating indicates corrosion resistance ability of the TiO_2_ nanotube and the mesoporous silica imparted by the epoxy coating.

**Figure 6:**
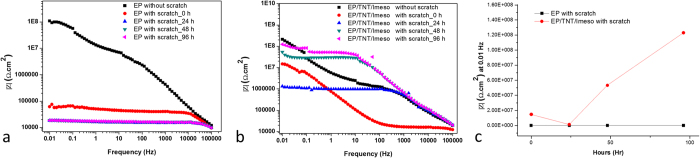
EIS Bode diagrams of (**a**) EP and (**b**) EP/TNT/Imeso coatings in 3.5 wt% NaCl solution at different immersion time, (**c**) variation of impedance value at 0.01 Hz with immersion time for both coatings.

The self-healing ability of the coatings was evaluated using an EIS study of the scratched samples at regular time intervals^3^,^9^. Cross-scratches were made on the coated samples using a scalpel. The corrosion resistances of coated samples with and without scratches were monitored for 5 days. Figure 6a and b shows the EIS bode plots for scratched EP and EP/TNT/Imeso coatings immersed in a 3.5 wt% NaCl solution. For comparison, the EIS bode plots for coatings without scratches are also included. It can be observed that the scratching led to a severe decline in the impedance value in the entire frequency region for both the control epoxy and EP/TNT/Imeso coatings. For the control epoxy coating, after an immersion time of 24 hours, the impedance curve dropped to its minimum value, and upon further immersion (48 and 96 h), the impedance curve remains its minimum value. However, in the case of the EP/TNT/Imeso coating, it can be observed after an initial reduction in impedance value, the impedance value began to increase in successive days. The variation in impedance value at 0.01 Hz with the immersion time for the control sample and the EP/TNT/Imeso coating is given in Fig. 6c. The impedance value at 0.01 Hz for the control epoxy coating remains same over the entire immersion time, whereas that of the EP/TNT/Imeso coating starts to increase after 48 hours. This observation confirmed the recovery of the anticorrosive property of the scratched coating during immersion time. It is calculated that the EP/TNT/Imeso coating recovered 57% of its anticorrosive property. The slow release of a sufficient amount of epoxy pre-polymer from the TiO_2_ nanotube lumen and its further crosslinking with the immobilized amine curing agent healed the scratched region. The slow release of the epoxy pre-polymer from TiO_2_ nanotubes, together with the slow gelation time required for the immobilized curing agent, caused a slow recovery of the scratched area in the EP/TNT/Imeso coating. In order to better understand the curing characteristics of the system, a control experiment was done with nanotube alone. The coating containing nanotube alone didn’t show any self-healing properties, which indicated that the cure reaction of epoxy monomer inside the nanotube takes place only when it comes to contact with the amine curing agent which is immobilized in mesoporous silica. The slow and steady release of the epoxy pre-polymer and its further cross-linking inside the scratch makes the EP/TNT/Imeso coating applicable for long-term corrosion resistance. However, the impedance response of the EP/TNT/Imeso coatings undergoing the healing process is different from the unscratched (EP/TNT/Imeso without scratch) and unhealed scratched (EP/TNT/Imeso with scratch_0 h and EP/TNT/Imeso with scratch_24 h) coatings. The impedance value in the middle frequency region is higher for the scratched EP/TNT/Imeso coatings undergoing the healing process (i.e., coatings after 48 hours of immersion) than that of the unscratched and unhealed scratched EP/TNT/Imeso coatings, indicating a high charge-transfer resistance of the healed coatings.

**Figure 7:**
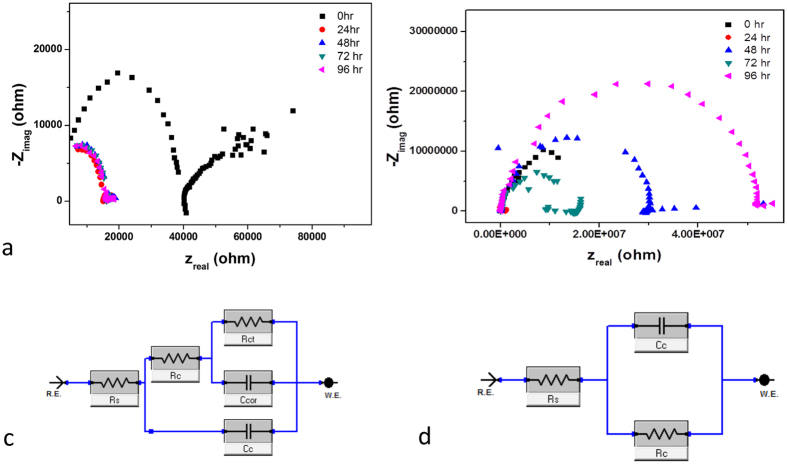
Nyquist plot of (**a**) scratched control epoxy coating and (**b**) scratched EP/TNT/Imeso coating at different immersion time: Equivalent circuits for (**c**) control epoxy coating without scratch and with scratch over the entire time intervals and EP/TNT/Imeso without scratch and with scratch at the initial time (0 hr) (**d**) for scratched EP/TNT/Imeso coating at the later stages of immersion (from 24 hr to 96 hr).

Further, the Nyquist plots for the scratched coating from 0 to 96 hours of immersion time are shown in Fig. 7a and b. The decrease in the radius of the semi-circles indicates the drop of the anticorrosion efficiency of the control epoxy coating from 24 hours of immersion time. However, in the case of EP/TNT/Imeso coating, the initial reduction at 24 hours begins to increase from 48 hours onwards, which indicating the recovery of the anticorrosive property during the immersion.

The electrical equivalent circuit used to fit the EIS plots for the control epoxy coating and the EP/TNT/Imeso coating is shown in Fig. 7c and d. The fitted circuit for the control epoxy coating without scratch and with scratch over the entire time intervals and the EP/TNT/Imeso coating without scratch and with scratch at the initial time (0 hr) are shown in Fig. 7c. Figure 7d shows the fitted circuit for the scratched EP/TNT/Imeso coating at the later stages of immersion (from 24 hr to 96 hr). In the models, Rs is the resistance of the electrolyte, Rc and Cc represent coating resistance and coating capacitance, respectively, and Rct and Ccor represent the charge transfer resistance and double layer capacitance, respectively. The values Rc and Rct obtained by fitting the EIS plots for the control epoxy coating and the EP/TNT/Imeso coating are listed in Table 1. It was observed that the immersion time increases the coating resistance of the scratched EP/TNT/Imeso coating, suggesting that the recovery of the scratched area reduces the penetration of the electrolyte. However, the Rc value for the control epoxy coating remains at the minimum value indicating an unrecovered scratch throughout the immersion time. The variation of the Rc value with the immersion time shows better agreement with the observed trend in the low frequency impedance value for the scratched coatings (Fig. 6c). The charge transfer resistance parameter (Rct), a measure of the resistance to the electron transfer across the metal surface^32^, is normally used to quantify the disbanding of the coatings and the onset of corrosion at the interface^33^. The higher value of Rct for EP/TNT/Imeso during the initial immersion period (0 h) shows that it is more difficult for the corrosion reaction to occur.

### Microscopic observation of self-healing process

**Figure 8:**
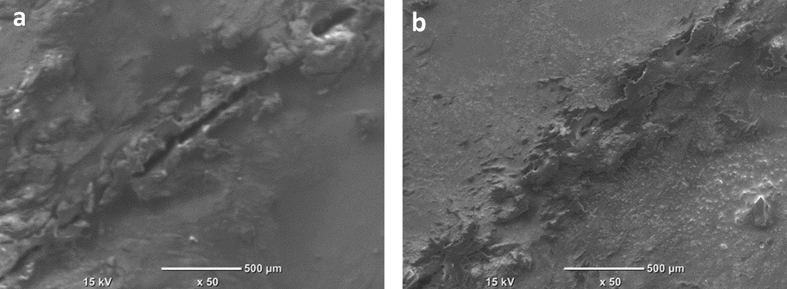
SEM images of the scratch on (**a**) 1^st^ day and (**b**) 5^th^ day in EP/TNT/Imeso self-healing coating.

The self-healing ability of the EP/TNT/Imeso coating was monitored using SEM. Figure 8 shows the SEM images of the scratched EP/TNT/Imeso coating at different time intervals. It is clear from the micrographs that the extent of scratch substantially decreased, and the damaged area was nearly healed within 5 days (96 hours), confirming the EIS results. This observation further confirms the release of the epoxy from the TiO_2_ nanotube into the scratch and its successive crosslinking upon contact with the immobilized amine in mesoporous silica. The larger size of the mesoporous silica facilitates more chance of contact with the epoxy pre-polymer released from the TiO_2_ nanotube upon crack.

## Conclusions

Mesoporous silica (SBA 15) with a high surface area and an average pore size of 4 nm and TiO_2_ nanotubes (TNT) with an average tube diameter of 10 nm were synthesized and effectively used as containers to load an epoxy pre-polymer and an amine curing agent. The epoxy pre-polymer was encapsulated inside the lumen of TiO_2_ nanotubes by a vacuum loading process. The amine curing agent was immobilized in mesoporous silica by shaking incubation. Epoxy coatings with both epoxy-encapsulated TiO_2_ nanotubes and amine immobilized mesoporous silica were prepared on carbon steel. The satisfactory filling of the TiO_2_ pores was confirmed by the estimated dead pore ratio of 94% for EP/TNT/Imeso coating composites. TEM images of the EP/TNT/Imeso coating composites showed the localization of the amine curing agent immobilized mesoporous silica among the groups of filled TiO_2_ nanotubes. The self-healing ability of EP/TNT/Imeso was confirmed by the EIS bode plots. EP/TNT/Imeso coating recovered 57% of its anticorrosive property in 5 days. The experimental EIS data were well-fitted with electrical circuits, and the subsequently extracted parameters were further used to confirm the self-healing ability of the EP/TNT/Imeso coating. The increase in coating resistance (Rpo) with the immersion time suggested the healing of the scratch in the EP/TNT/Imeso coatings. The self-healing process was monitored using SEM. The self-healing of the EP/TNT/Imeso coating has been proposed to occur by the release of the epoxy pre-polymer from the TiO_2_ nanotubes and its further crosslinking with the amine curing agent immobilized in the mesoporous silica. The slow and steady recovery of the damage makes this coating suitable for long term corrosion resistance applications. This self-healing technology could be extended to epoxy composites used in structural and aircraft parts. Further studies of the detailed kinetics of the healing process are in progress.

## Additional Information

**How to cite this article**: Vijayan P., P. and Al-Maadeed, M. A. S. A. TiO_2_ nanotubes and mesoporous silica as containers in self-healing epoxy coatings. *Sci. Rep.* **6**, 38812; doi: 10.1038/srep38812 (2016).

**Publisher's note:** Springer Nature remains neutral with regard to jurisdictional claims in published maps and institutional affiliations.

## Figures and Tables

**Table 1:** The fitting parameters obtained for scratched and unscratched control epoxy coatings.

Coating	Time (h)	Rc (ohms)	Rct (ohms)
EP without scratch		91.97 × 10^6^	5.18 × 10^6^
EP with scratch	0	19.54 × 10^3^	37.04 × 10^3^
	24	1.87 × 10^3^	15.06 × 10^3^
	48	1.63 × 10^3^	16.31 × 10^3^
	96	1.79 × 10^3^	16.02 × 10^3^
EP/TNT/Imeso without scratch		245.70 × 10^6^	1.37 × 10^6^
EP/TNT/Imeso with scratch	0	17.02 × 10^6^	4.30 × 10^6^
	24	963.9 × 10^3^	—
	48	29.36 × 10^6^	—
	96	55.18 × 10^6^	—
